# Large-Scale Spatial Distribution Patterns of Gastropod Assemblages in Rocky Shores

**DOI:** 10.1371/journal.pone.0071396

**Published:** 2013-08-13

**Authors:** Patricia Miloslavich, Juan José Cruz-Motta, Eduardo Klein, Katrin Iken, Vanessa Weinberger, Brenda Konar, Tom Trott, Gerhard Pohle, Gregorio Bigatti, Lisandro Benedetti-Cecchi, Yoshihisa Shirayama, Angela Mead, Gabriela Palomo, Manuel Ortiz, Judith Gobin, Adriana Sardi, Juan Manuel Díaz, Ann Knowlton, Melisa Wong, Ana C. Peralta

**Affiliations:** 1 Departamento de Estudios Ambientales and Centro de Biodiversidad Marina, Universidad Simón Bolívar, Caracas, Miranda, Venezuela; 2 School of Fisheries and Ocean Sciences, University of Alaska Fairbanks, Fairbanks, Alaska, United States of America; 3 Departamento de Ecología, Pontificia Universidad Católica de Chile, Santiago de Chile, Chile; 4 Suffolk University, Boston, Massachusetts, United States of America; 5 The Huntsman Marine Science Centre, St. Andrews, New Brunswick, Canada; 6 Centro Nacional Patagónico, Puerto Madryn, Chubut, Argentina; 7 Dipartimento di Biologia, University of Pisa, Pisa, Tuscany, Italy; 8 Seto Marine Biological Laboratory, Field Science Education and Research Center, Kyoto University, Shirahama, Wakayama, Japan; 9 University of Cape Town, Cape Town, Western Cape, South Africa; 10 Laboratorio de Ecosistemas Costeros, Museo Argentino de Ciencias Naturales “Bernardino Rivadavia”, Buenos Aires, Argentina; 11 Centro de Investigaciones Marinas, Universidad de La Habana, La Habana, Cuba; 12 Department of Life Sciences, The University of The West Indies, St. Augustine, Trinidad and Tobago; 13 Universidad Nacional de Colombia, Bogotá, Colombia; 14 Bedford Institute of Oceanography, Dartmouth, Nova Scotia, Canada; Bangor University, United Kingdom

## Abstract

Gastropod assemblages from nearshore rocky habitats were studied over large spatial scales to (1) describe broad-scale patterns in assemblage composition, including patterns by feeding modes, (2) identify latitudinal pattern of biodiversity, i.e., richness and abundance of gastropods and/or regional hotspots, and (3) identify potential environmental and anthropogenic drivers of these assemblages. Gastropods were sampled from 45 sites distributed within 12 Large Marine Ecosystem regions (LME) following the NaGISA (Natural Geography in Shore Areas) standard protocol (www.nagisa.coml.org). A total of 393 gastropod taxa from 87 families were collected. Eight of these families (9.2%) appeared in four or more different LMEs. Among these, the Littorinidae was the most widely distributed (8 LMEs) followed by the Trochidae and the Columbellidae (6 LMEs). In all regions, assemblages were dominated by few species, the most diverse and abundant of which were herbivores. No latitudinal gradients were evident in relation to species richness or densities among sampling sites. Highest diversity was found in the Mediterranean and in the Gulf of Alaska, while highest densities were found at different latitudes and represented by few species within one genus (e.g. *Afrolittorina* in the Agulhas Current, *Littorina* in the Scotian Shelf, and *Lacuna* in the Gulf of Alaska). No significant correlation was found between species composition and environmental variables (r≤0.355, p>0.05). Contributing variables to this low correlation included invasive species, inorganic pollution, SST anomalies, and chlorophyll-a anomalies. Despite data limitations in this study which restrict conclusions in a global context, this work represents the first effort to sample gastropod biodiversity on rocky shores using a standardized protocol across a wide scale. Our results will generate more work to build global databases allowing for large-scale diversity comparisons of rocky intertidal assemblages.

## Introduction

It has been long and generally recognized that the diversity of coexisting species has a fundamental influence on many ecological processes, including those processes that determine the stability of the community itself [1]–[2]. However, no general consensus has been reached on the “shape” or “characteristics” of this relationship (diversity-function); because, among many other reasons, the observed shape of the relationship depends on the scale of the observation. Consequently, any understanding of this relationship has to depart from a proper description of the distribution patterns of diversity across different spatial and temporal scales [3]–[9]. Moreover, it has also been shown, that the shape of the relationship might be subjected to anthropogenic influences operating at different spatial scales [10]. Insight of how species assemblages are established and the processes that shape their patterns of biodiversity is critical for understanding various aspects of global change. The impacts of global change range from climate effects on community structure, productivity and nutrient cycling to human-induced effects such as fishing pressure and the introduction of non-native species, although the latter can also occur through the extension of species distribution range in response to climate (e.g., [11]–[15]).

Even though many studies have been done on describing patterns of spatial and temporal distribution of species at small scales, those at large scales pose various challenges, as they cannot be easily extrapolated from models developed at small, local scales (e.g., [16]–[18]).

At large spatial scales, it has long been accepted that one of the most invariant patterns of biodiversity is the latitudinal cline of species richness, and consequently that ecological processes or factors associated to the latitudinal gradient (i.e. temperature, harshness) would be the key factors on determining patterns of spatial distribution of diversity at large spatial scales [19]–[24]. Although there are a few studies that either support or reject this pattern (especially in marine systems), these may not be comparable because they were focused on different geographical areas, with different sampling efforts, and taxonomic resolution. Therefore, a standardized approach, including a standardized protocol is needed. The implementation of such standard protocol would allow for a large scale analysis of taxon groups and habitat types improving our understanding of mechanisms underlying dynamics of a taxon assemblage, and providing the basis for new hypotheses. As a response to the need of data standardization for a better understanding of diversity patterns [23], [25], [26], the NaGISA Natural Geography in Shore Areas, a field program of the Census of Marine Life project, was implemented in 2003 as a global initiative to study coastal diversity, distribution, and abundance by using a standardized protocol in shallow marine habitats. By 2010, NaGISA had sampled more than 250 sites within 28 globally distributed countries, of which 182 sites were rocky shores. Analysis of data collected under this scheme has shown that patterns of distribution of diversity and biomass of various taxonomic groups (e.g. macroalgae, decapods and echinoderms) are very complex and not always follow the expected latitudinal gradient of species diminution towards the poles [27]–[31], or appear to be superseded by regional diversity hotspots [29]. The NaGISA database has also allowed correlation analyses between species diversity or composition and environmental-anthropogenic variables at large spatial scales, showing that potential drivers of diversity at large scale vary depending on the group being analysed [27]–[31]. Given this variability of outcomes for different taxonomic groups, this study will focus on gastropods, one of the most diverse and ecologically important groups in the rocky shore environments.

Gastropods are an important and representative component of rocky shore assemblages [32]–[35]. They are the most species rich class within the mollusks with a reasonably well-known taxonomy [36]–[38], and has been, after fishes, the most studied group in marine systems, so there is an extensive ecological literature to compare results with. The gastropods were one of the first groups in which a clear latitudinal cline in benthic marine species richness was observed [39], [40], a trend that was re-confirmed in more recent studies [41]–[43], but not demonstrated in the southern hemisphere [25] or in any case, different from the northern hemisphere [44], [45]. Most of these studies were carried out by analyzing local and regional species lists compiled from all marine ecosystems, however such regional inventories are known to be incomplete even in the best sampled regions [46]. Regions with the highest number of gastropod species are the waters surrounding Japan [47] and Australia [48] with more than 6000 species, while the poorest regions in terms of species richness are the Canadian Arctic [49] and the Tropical West Atlantic with less than 210 species [50]. The problem of determining species diversity and furthermore, abundance, is even more critical in lesser-known regions due to severe restrictions in sampling efforts (e.g. number of samples, ecosystems sampled, lack of standardization in collection methods), taxonomic capacity and expertise, and general resources to support this type of research [46], [51].

Therefore, this study aims to describe diversity and abundance distribution patterns of gastropods from near shore rocky habitats and to identify possible drivers that might be related such patterns. Description of these patterns is the first necessary step to then propose specific hypotheses about specific drivers for these assemblages. For this, we used the NaGISA dataset to (1) describe broad scale patterns of gastropod diversity and abundance, including patterns based on feeding modes, (2) evaluate the existence of latitudinal pattern of gastropod richness, abundance and/or regional hotspots, and (3) identify environmental and anthropogenic drivers that may explain large scale patterns of these assemblages.

## Materials and Methods

### Sampling

**Figure 1:**
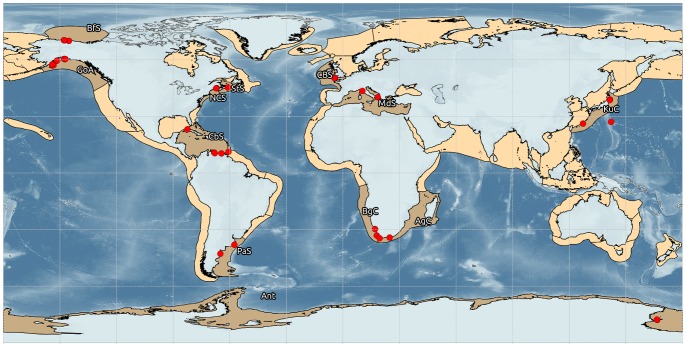
Global distribution of the sampling sites within the Large Marine Ecosystems (LME). BfS: Beaufort Sea; GoA: Gulf of Alaska; CBS: Celtic-Biscay Shelf; StS: Scotian Shelf; NCS: North East US Coast Shelf; KuC: Kuroshio Current; MdS: Mediterranean Sea; CbS: Caribbean Sea; AgC: Agulhas Current; BgC: Benguela Current; PaS: Patagonian Shelf.

Gastropod diversity and abundance were estimated using the NaGISA standardized protocol developed for the Census of Marine Life program [34]. Gastropod surveys were done at 45 widely distributed rocky shore shallow sites and grouped within 12 Large Marine Ecosystems (LMEs) as defined by the National Oceanic and Atmospheric Administration (NOAA) [52] to allow for large-scale comparisons. Selection of sites was based, as much as possible, on relatively pristine conditions and remoteness from direct human influence. However, within each LME, site selection was biased by accessibility and location of contributing investigators, resulting in an unbalanced distribution of sampling localities across latitude and longitude. Most samples were collected in the northern and western hemispheres (Table 1). Between one and 11 sites were sampled in each LME. Although this sampling size is clearly an under-representation of each LME [27]–[31], it still allows for comparisons of larger-scale patterns above the local variability (Figure 1). Within each site, five replicate 0.0625 m^2^ quadrats (25×25 cm^2^) were sampled randomly distributed along a 30–50 m transect at the high, mid, and low intertidal strata and at 1, 5, and 10 m (when available) depth in the subtidal. The epibenthic assemblage was removed from the quadrat area, sieved over 500 µm mesh, and gastropods were sorted and identified to the lowest taxonomic level possible (species in most of the cases). Sampling took place between June 2004 and January 2009. Since sampling of the sites did not take place at the same time, no analysis on temporal variation was carried out, however, to diminish the effects of temporal (i.e. seasonal) variation on our spatial analyses, we selected from the database the data corresponding to the warmer season for each site.

**Table 1:** Location of the 45 sampled sites in each of the twelve LME’s.

LME	Abbreviation	Ocean	Country	Site Name	Latitude	Longitude
**Beaufort Sea**	**BfS**	Arctic	USA	DS11	70.322	−147.579
				DS4	70.032	−145.269
				DSC	70.025	−145.253467
				DSE	70.026	−145.258833
				DSW	70.025	−145.25755
				E1	70.315	−147.732
				E2	70.318	−147.715
				E3	70.325	−147.649
				W1	70.370	−147.873
				W2	70.370	−147.860
				W3	70.376	−147.794
**Gulf of Alaska**	**GoA**	Pacific	USA	Akhiok Bay	56.947	−154.12925
				Cohen Island	59.547	−151.547222
				Elephant Island	59.547	−151.513889
				Green Island	60.300	−147.412222
				Knight Island	60.484	−147.735556
				Montague Island	60.391	−147.121667
				Old Harbor	57.157	−153.388683
				Outside Beach	57.157	−153.388683
				Uyak bay	57.574	−154.111944
**Celtic-Biscay Shelf**	**CBS**	Atlantic	UK	Batten Bay	50.357	−4.127
				Looe	50.341	−4.460
**Scotian Shelf**	**StS**	Atlantic	Canada	Canso	45.323	−60.965
**Northeast U.S**	**NCS**	Atlantic	Canada	Simpsons Island	45.004	−66.914
**Continental Shelf**			USA	Birch Island	44.871	−67.150
				Outer Birch Island	44.871	−67.150
**Kuroshio Current**	**KuC**	Pacific	Japan	Ksen-numa	38.897	141.62495
				Maenohama	26.187	127.281
				Sakamoto	38.645	141.477
				Suzaki	27.074	142.188
**Mediterranean**	**MdS**	Mediterranean	Italy	Calafuria	43.473	10.333
				Torre del Serpe	40.145	18.505
**Caribbean Sea**	**CbS**	Atlantic	Cuba	Playa de 16	23.128	−82.422
			Trin. & Tob.	Fort Granby	11.186	−60.661
			Venezuela	Cayo Mero	10.820	−68.248
				Punta Tigrillo	10.380	−64.395
				Punta Yapascua	10.475	−67.901
**Benguela Current**	**BgC**	Atlantic	South Africa	Brasil	−29.718	17.058
				Kreef Bay	−33.143	17.982
				Pearly Beach	−34.585	19.457
**Agulhas Current**	**AgC**	Indian	South Africa	Oyster Bay	−34.199	24.798
**Patagonian Shelf**	**PaS**	Atlantic	Argentina	Playa Chica	−38.020	−57.520
				Punta Este	−42.787	−64.954
**Antarctic**	**Ant**	Southern	USA	Daytons Wall	−77.853	166.661
				Evans Wall	−77.657	166.518

All necessary permits were obtained for the described field studies: University of Pisa and Council of Livorno, Italy (Mediterranean sites), Department of Fisheries and Oceans, Canada (Canso and Simposon Island sites), Alaska Department of Fish and Game (Gulf of Alaska sites: Kachemak Bay - National Estuarine Research Reserve-, Outside Beach, Cohen Island, and Elephant Island), Maine Department of Marine Resources (Birch Island/Outer Birch Island sites), Oficina Administrativa de Permisiones del Ministerio del Poder Popular del Ambiente, Venezuela (Venezuelan sites – National Parks-), and South African Department of Environmental Affairs (South African sites). Antarctic samples were collected under the umbrella of the United States Antarctic Program (USAP) at McMurdo Antarctic Station. For the rest of the sites, no specific permits were required for the described field studies as the locations were not privately-owned or protected in any way, and the field studies did not involve endangered or protected species.

### Environmental Data

To link the gastropod assemblages in rocky shores with environmental variables, 15 environmental variables considered either as “natural” or as “anthropogenic” (Table 2) were examined to test if these could be important drivers of gastropod diversity and abundance associated with these ecosystems. Variables grouped as “natural” and not directly related to human activities were sea-surface temperature (SST), sea-surface temperature anomalies (SSTa), chlorophyll-a (CHA), chlorophyll-a anomalies (CHAa), rainfall (RAI), rainfall anomalies (RAIa), photoperiod (PHO), and mean value of tidal amplitude (TID). SST and CHA data were provided by the MODIS Aqua mission, RAI data were compiled using the TOVAS web-based application, PHO was calculated as the difference between the sunrise and sunset time [53], and TID was calculated using the Program WXTide32 Version 4.7. Anomalies for each of these variables were defined as the numbers of events that surpassed two standard deviations of the average for each of those variables for a given year at any given location. Variables grouped as “anthropogenic” or directly related to human activities were inorganic pollution (INP), organic pollution (ORP), acidification (AC), incidence of invasive species (INV), human population pressure (HUM), shipping activity (SH), and ocean-based pollution (OBP) using the categories provided by Halpern et al. [54] (see more specifics in Table 2). Since environmental data could not always be collected or predicted from the exact sampling sites, and there was some inaccuracy of satellite-derived data from optical sea-surface properties (e.g., CHA) at small spatial scales [54], we used the LME scale to allow for the interpretation of large-scale variability. While the nearshore is a notably variable environment at the local scale, large-scale variability has reported to be even higher [27], [28].

**Table 2:** List and sources of the natural and anthropogenic variables used in the analysis.

Variable	Abbreviation	Description/Source/Reference
**Natural**		
Sea surface temperature	SST	Average of monthly values of the MODIS Aqua mission from July 2002 to December 2009
Sea surface temperature anomalies	SSTa	Numbers of events that surpassed 2 standard deviations of the average temperature for a given year
Chlorophyll-a	CHA	Average of monthly values of the MODIS Aqua mission from July 2002 to December 2009
Chlorophyll-a anomalies	CHAa	Numbers of events that surpassed 2 standard deviations of the average chlorophyll-a for a given year
Rainfall	RAI	Average of monthly accumulated rainfall from January 1979 through September 2009 obtained using the TOVAS web-based application
Rainfall anomalies	RAIa	Numbers of events that surpassed 2 standard deviations of the average rainfall for a given year
Photoperiod	PHO	Common astronomical formulae were used to compute the difference between sunrise and sunset times (Meeus, 1991)
Tides	TID	The tidal amplitude was calculated as the mean value for the sampled month. We used the Program WXTide32 Version 4.7 (2007). If there was no information for the desired location, we used the closest location based on the coordinates.
**Anthropogenic**		
Inorganic pollution	INP	Urban runoff estimated from land-use categories, US Geologic Survey (http://edcsns17.cr.usgs.gov/glcc/) (Halpern et al., 2008)
Organic pollution	ORP	FAO national pesticides statistics (1992–2001), (http://faostat.fao.org) (Halpern et al., 2008)
Nutrient contamination	NUTC	FAO national fertilizers statistics (1993–2002), (http://faostat.fao.org) (Halpern et al., 2008)
Acidification	AC	Aragonite saturation state 1870–2000/2009, 1 degree lat/long resolution (Halpern et al., 2008)
Invasive species incidence	INV	Cargo traffic 1999–2003 (Halpern et al., 2008)
Population pressure	HUM	Estimated as the sum of total population adjacent to the ocean within a 25 km radius of the sampling site. LandScan 30 arc-second population data of 2005 were used (Halpern et al., 2008)
Shipping activity	SH	Commercial ship traffic 2004–2005 (Halpern et al., 2008)
Ocean-based pollution	OBP	Modeled as a combination of commercial shipping traffic data and port data (Halpern et al., 2008)

Environmental variables related to direct anthropogenic influences were collected at 1 km resolution. When a site was within 50 km of the model, a spline interpolation was used to the raster data to compute the variable value at the coordinate of the sampling site.

### Data Analyses

To provide a local estimate of taxon richness we combined the data from the intertidal and subtidal strata for each site. Given that the sampling effort between LMEs was unbalanced in the number of samples (e.g. 10 sampling units in Agulhas Current vs 308 sampling units in the Gulf of Alaska), we standardized the number of taxa at each site with different sample numbers. For this, we used saturation curves following the Ugland-Gray-Ellingsen or UGE method [55], which estimates how many taxa would have been found at each site if a specific number of sampling units ( = quadrats) had been sampled at each site (for 999 permutations). Here, we standardized the analysis for an arbitrary sampling size of 10 replicates [56], so only those sites in the NaGISA database in which 10 or more sampling units across all depth and intertidal levels had been sampled were considered in the analyses. The number of sites within one LME varied between 1 (Agulhas Current and Scotian Shelf) to 11 (Beaufort Sea). To detect possible patterns of species distribution across different latitudes, a Pearson correlation analysis was done between taxon richness and abundance per site and latitude. Average densities of total gastropod taxa were standardized for the total area sampled at each site and scaling those data to a standard 0.0625 m^2^ area (a 25×25 cm^2^ quadrat). Additionally, we also searched for patterns of the most widely distributed families, which in this work, we considered as those that were present in four or more LME’s.

To examine geographical patterns of community similarity, we used multivariate methods [57]–[59] and the Primer 6.1.3 (Plymouth Routines in Multivariate Ecological Research) Permanova+software package. We transformed the taxon composition data per site into a presence-absence matrix, which was then used to construct a similarity matrix based on the taxonomic dissimilarity coefficient *Theta* [60], [61]. *Theta* is a Presence/Absence (P/A) measure (similar to Kulczynski’s) but that takes into consideration the distance (*w*) through the taxonomic tree from species *i* of sample 1 to species *j* of sample 2 (see [61] for *Theta* equation). The *Theta* coefficient allows for comparison of samples across large geographical scales that do not share many species and also considers the taxonomic relationship of species found in each sample [61]. To visualize the distances among centroids of sampling sites [59], we performed a Canonical Analysis of Principal Coordinates (CAP) ordination [58], in which the LME is considered the predictor variable that creates in *a priori* fashion the taxonomic differences in the data.

To analyze if taxon composition patterns of gastropod assemblages were correlated to environmental drivers, we created a variable-by-site matrix with variables normalized to a common scale. To detect possible effects of distances among sampling sites, geographic coordinates were included in this matrix for further analyses. Co-linearity was examined prior to analyses to avoid using redundant data. Redundant environmental variables and proper transformations of the data were identified using multiple correlation analysis (draftsman plots) after square-root transformation of skewed variables and excluded from the analysis. To select the combination of variables that best explained the observed biological distribution patterns, a similarity matrix of environmental variables based on Euclidean distances was linked to the taxonomic dissimilarities patterns (*Theta* matrix) among LMEs using the BEST routine BIOENV [62] from the PRIMER-E [63] with PERMANOVA [59] software.

## Results

**Figure 2:**
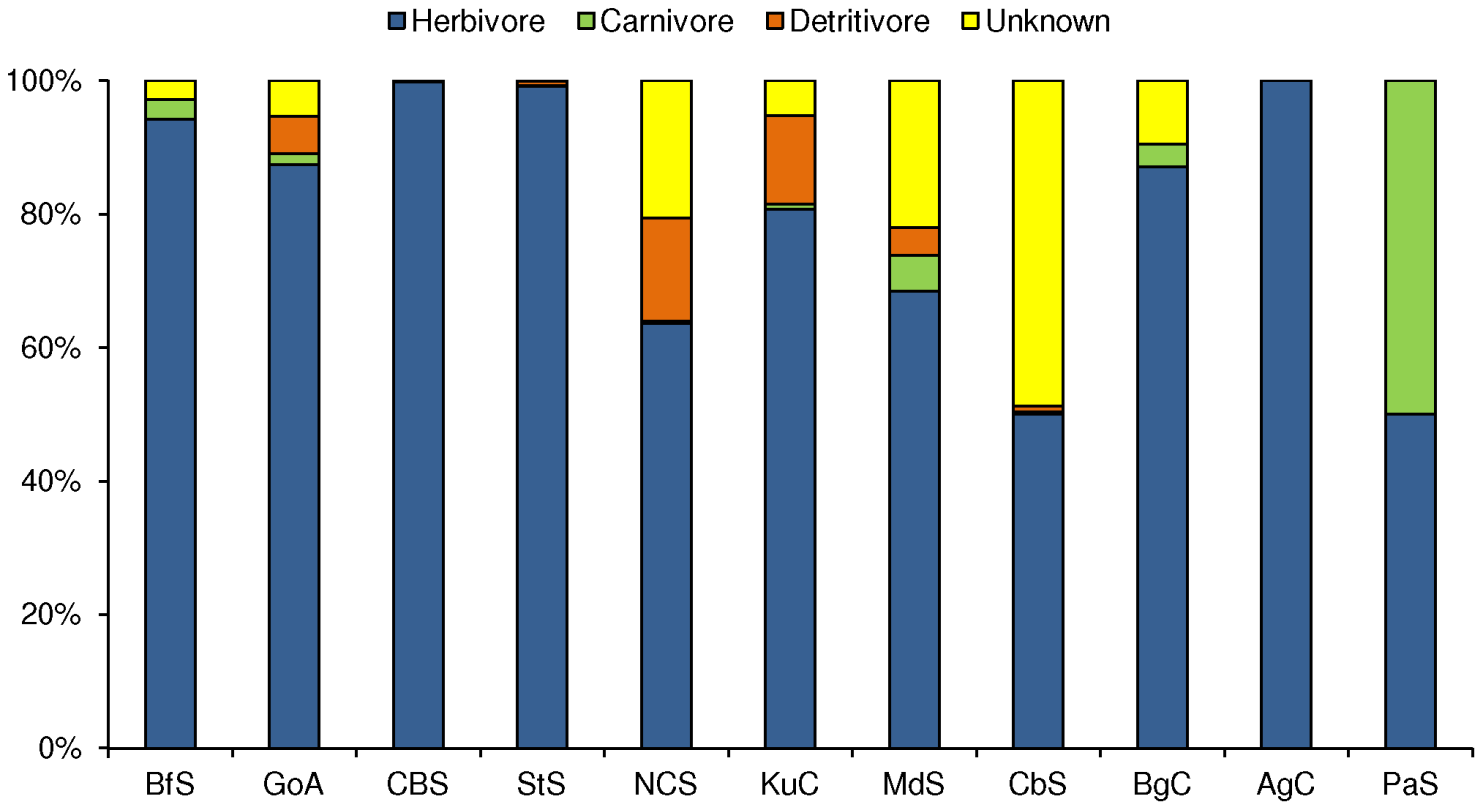
Proportional abundance of gastropod functional feeding groups per LME. LME abbreviations as in figure 1.

A total of 393 gastropod taxa were collected within 87 families (Table S1). Nearly 14% of these families were represented by 10 or more species, while the majority of the families (86%) were represented by 1 to 9 species. The richest families in the overall dataset in terms of number of species were the Trochidae and the Rissoidae with 32 and 30 species, respectively, followed by the Lottiidae (24 species), Littorinidae (22), Muricidae (21), Fissurellidae (17), Collumbellidae (16), Patellidae and Buccinidae (15 species each), Conidae (12), and Cerithiidae and Pyramidellidae (10 species each). None of the species found at each of the sites are listed as alien/invasive for their particular locality except for *Littorina littorea* which has been reported as an early introduced species in the Western North Atlantic [64]. In all LMEs, assemblages were dominated by few species and most other species were rare (Table 3). In terms of trophic groups, herbivores were the most diverse and abundant in all LMEs (Figure 2). Carnivorous gastropods were rare in all LMEs (<5% when present), and a considerable number (30%–40%) of gastropods species with unknown trophic preferences were found in the Kuroshio Current, Mediterranean Sea and Caribbean Sea LMEs. In the Antarctic, only one of the three taxa found was identified to species level (*Tritonia challengeriana*: carnivore).

**Table 3:** Dominant gastropod species or taxa (that amounted to 60% of total densities) for each LME (LMEs ordered by UGE value).

LME	NS (sites)	NQ (quadrats)	S (taxa)	UGE (n = 10)	Dominant species
Beaufort Sea	11	175	20	4	*Boreocingula martin*
Gulf of Alaska	9	308	100	31	*Lacuna vincta, Littorina sitkana, Cingula katherinae, Lacuna sp.*
Celtic-Biscay Shelf	2	28	9	7	*Gibbula umbilicales, Littorina obtusata, Littorina littorea*
Scotian Shelf	1	25	16	11	*Littorina sp.*
Northeast U.S. Continental Shelf	3	118	61	18	*Onoba aculeus, Skeneopsis planorbis, Littorina obtusata*
Kuroshio Current	4	16	30	22	*Cerithidae, Nodilittorina sp, Monodonta labio*
Mediterranean Sea	2	35	100	66	*Rissoa similis, Bittium latreilli, Pisinna glabrata, Bittium reticulatum, Skeneopsis planorbis, Pusillina philippi*
Caribbean Sea	5	55	62	19	*Planaxis lineatus, Vermetidae sp., Sinezona confusa*
Benguela Current	3	43	26	17	*Afrolittorina africana, Patella granularis, Helcion dunkeri*
Agulhas Current	1	10	5	5	*Afrolittorina knysnaensis*
Patagonian Shelf	2	50	6	6	*Siphonaria lessoni, Tegula patagonica*
Antarctic	2	13	3	2	*Tritonia challengeriana*

Includes number of sites (NS), number of sampled quadrats (NQ), total number of observed taxa (S), estimators of number of taxa for a standard sampling size of 10 quadrats based on saturation curves (UGE method), and most common species or taxa per LME. LMEs arranged from north to south.

**Figure 3:**
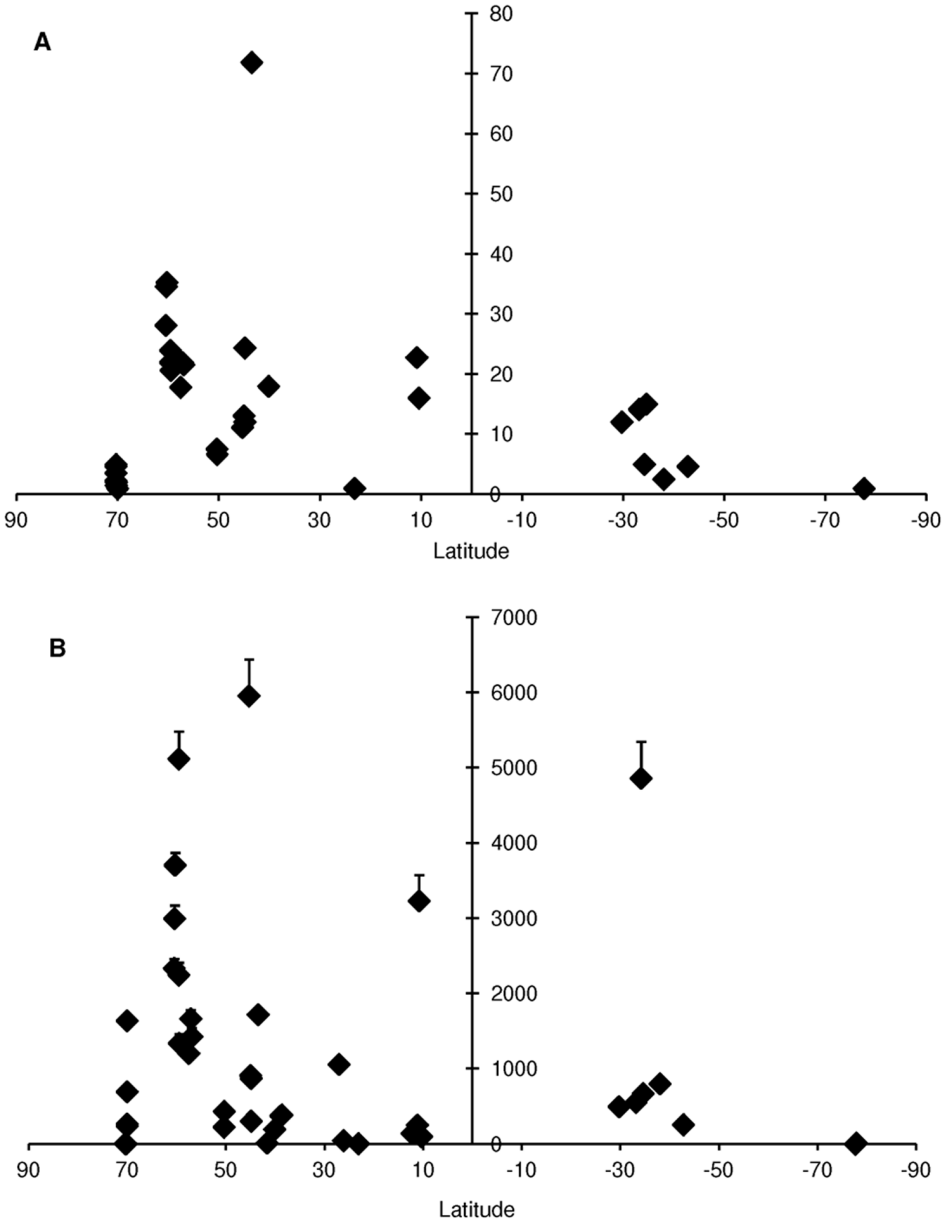
Species richness using UGE estimates (n = 10) (A) and abundance (ind/m^2^) (B) across latitude. LME abbreviations as in figure 1.

**Figure 4:**
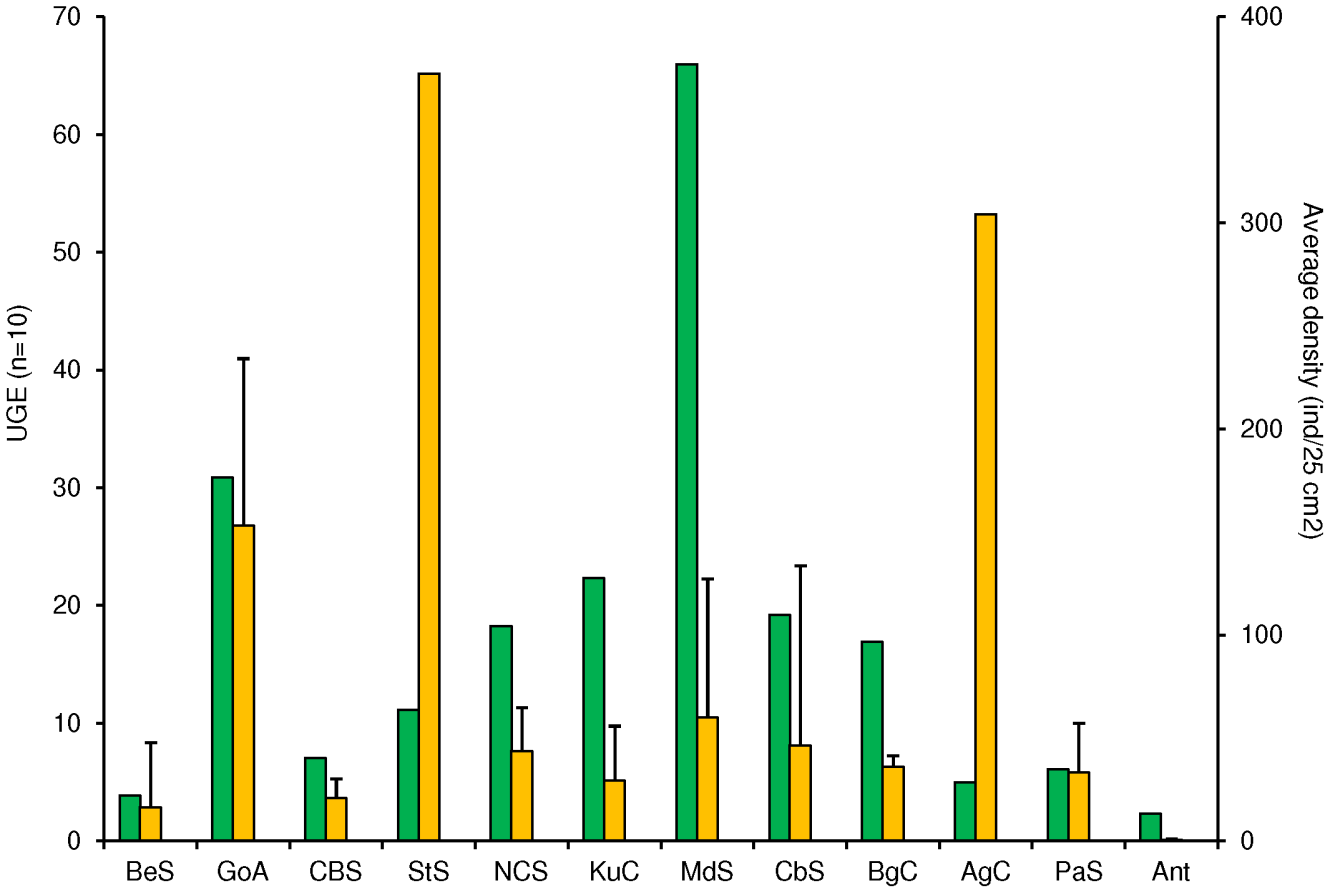
Comparison of the estimated species richness (UGE, n = 10) and average density (ind/0.0625 m^2^ = ind/quadrat of 25×25 cm^2^) among the LMEs. Yellow bars indicate average density values and green bars represent standardized (UGE) number of species. LME abbreviations as in figure 1.

The Pearson correlation test did not support a relationship between latitude and the UGE standardized estimate of species richness (r = 0.16, T = 0.9675, p>0.05). In fact, a greater dispersion of standardized richness was observed among sites within similar latitudes than across latitude (Figure 3A). For example, at 42°–45° N, sites with either high (Calafuria in the Mediterranean, UGE = 71.9 for n = 10) and low (Canso in the Scotian Shelf, UGE = 11.0 for n = 10) estimates of standardized richness were found. Similarly, no latitudinal trend was observed in terms of gastropod average densities (Pearson r = 0.077, T = 0.52, p>0.05). Sites with high densities were observed near 60°N (Elephant, Knight, and Montague Islands), 45°N (Canso), 10°N (Cayo Mero) and 34°S (Oyster Bay) (Figure 3B). At these latitudes, most of the high densities observed resulted from a few species within one or two genera, and all of the family Littorinidae with the exception of the Caribbean sites at 10°N, in which the most abundant species were *Angiola lineata* (Family Planaxidae) and *Sinezona confusa* (Family Scissurellidae). Within the Littorinidae, two species of the genus *Afrolittorina* were found with an average density of 4864±480 ind/m^2^ in the Agulhas Current (Oyster Bay, 34°S), while on the Scotian Shelf (Canso, 45°N), the genus *Littorina* was the most abundant with an average density of 5955±485 ind/m^2^, and in the Gulf of Alaska, the genus *Lacuna* was the most abundant with an average density of 3706±163 ind/m^2^. Individuals of the littorinid family accounted for 99% of all abundance in the Agulhas Current and Scotian Shelf LMEs and for nearly 60% of all abundance in the Gulf of Alaska. When examining standardized richness estimates at the LME scale, the highest estimates for species richness were found in the Mediterranean Sea (66.0 for n = 10) and the Gulf of Alaska (30.9 for n = 10) (Figure 4). Low richness (UGE <10) was found in some of the cold-water LMEs, such as the Beaufort Sea Shelf and the Celtic-Biscay Shelf in the northern hemisphere, and the Patagonian Shelf and Antarctica in the southern hemisphere, but also in the warmer Agulhas Current (Table 3). There was no evident trend between species richness and abundance. For example, LMEs such as the Scotian Shelf and Agulhas Current, which were among those with the lowest UGE estimates for species richness, had the highest average gastropod densities, while the highly diverse Mediterranean Sea had relatively low densities.Both polar LMEs, the Beaufort Sea and the Antarctic, had low estimates for species richness and densities, while the relatively taxon rich Gulf of Alaska, had intermediate density levels (Figure 4).

**Figure 5:**
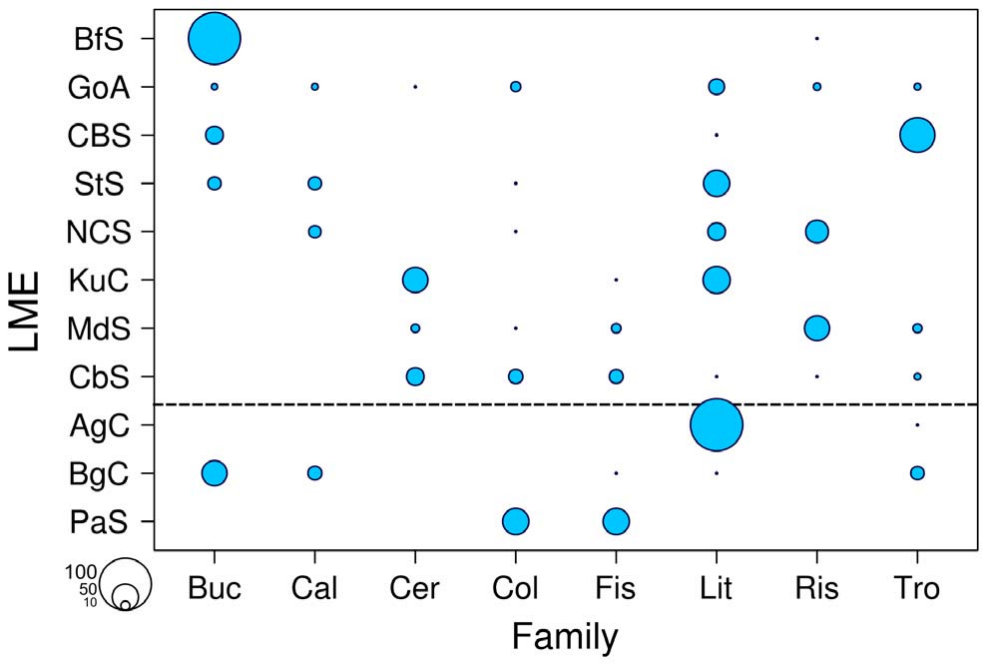
Relative contribution of the number of species (UGE standardized for n = 10) within the eight cosmopolitan families among the LMEs. Buc: Buccinidae, Cal: Calyptraeidae, Cer: Cerithiidae, Col: Columbellidae, Fis: Fissurellidae, Lit: Littorinidae, Tro: Trochidae, Ris: Rissoidae. LME abbreviations as in figure 1. Horizontal line separates northern from southern hemispheres.

Eight out of the 87 families reported in this study (9.2%) appeared in four or more different LMEs (Figure 5). The most widely dispersed families comprised the Littorinidae (in 8 LMEs), the Columbellidae and Trochidae (in 6 LMEs), the Buccinidae, Fissurelidae, and Rissoidae (in 5 LMEs), and the Calyptraeidae and Cerithidae (in 4 LMEs). Two of these eight families (25%), were restricted to the northern hemisphere (Cerithiidae and Rissoidea), while no family was exclusive to the southern hemisphere and none appeared in the Antarctic region. The pattern of the standardized measure of richness was not homogenous throughout the LMEs for these widely dispersed families. For example, species of the family Buccinidae accounted for most of the standardized richness measure in the Beaufort Sea in the northern hemisphere, while species of the Littorinidae and Columbellidae families contributed more equally to the total diversity across LMEs. Species of the Trochidae family also contributed relatively equally across LME’s with the exception of the Celtic-Biscay Shelf, where the species diversity of this family represented an important contribution to its total diversity.

**Figure 6:**
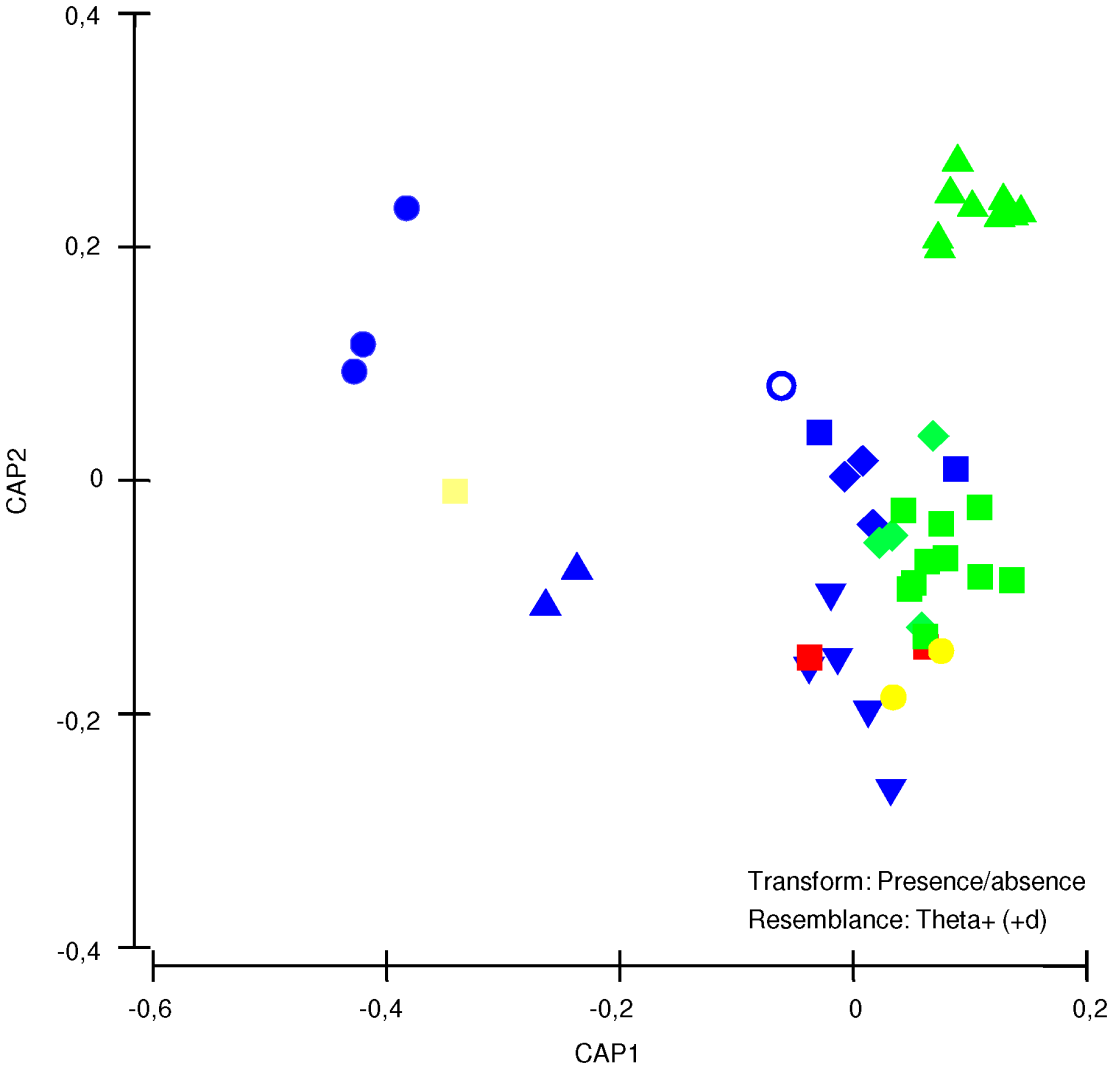
Canonical analysis of principal coordinates (CAP) plots generated from taxonomic dissimilarity coefficients (theta) of the biological data matrix, using LMEs as predictor factor. Blue circle = Benguela Current, Blue triangle = Celtic-Biscay Shelf, Yellow square = Agulhas Current, Hollow blue circle = Scotian Shelf, Inverted blue triangle = Caribbean Sea, Green Triangle = Gulf of Alaska, Blue square = Patagonian Shelf, Yellow circle = Antarctic, Red square = Mediterranean Sea, Blue diamond = Northeast US Continental Shelf, Green square = Beaufort Sea, Green diamond = Kuroshio Current.

A constrained ordination (CAP) of sampling sites using LME as a predictor factor, effectively showed that some LMEs were distinctly different based on the taxonomic dissimilarity of the assemblages (Fig. 6). For example, the Agulhas Current, Benguela Current and Celtic-Biscay Shelf LMEs were most separated from all other LMEs along the first axis (CAP1 = 98.2%), indicating very different taxonomic structure of species assemblages. Similarly, the Gulf of Alaska showed a distinct separation from the rest over the second axis (CAP2 = 96.8%). Most LMEs, however, were not clearly different in terms of their taxonomic structure when higher taxonomic hierarchies were included in the analysis (genera, families, and order). Interestingly, some LMEs with no species in common such as: the Caribbean Sea and the Beaufort Sea (Table S1), had a very similar taxonomic structure. Similarly to the previous univariate analyses (standardized UGE and total densities), the CAP results showed no latitudinal gradient in terms of the taxonomic structure of the gastropod assemblage.

There was no significant correlation between environmental and biological (taxonomic composition) matrices by means of a BIOENV routine (ρ = 0.355, p>0.05) (Table 4). Variables responsible for this low correlation were: INV, INP, SSTa, and CHAa. Further combinations, of fewer variables or including rainfall anomalies, explained the biological data with a slightly lower correlation index.

**Table 4:** Bio-ENV results showing the environmental variable combinations that best match the biotic similarity matrices using the weighted Spearman rank correlation (ρ) (p>0.05).

Number of Variables Considered	Correlation Coefficient (ρ)	Selection
4	0.355	INV, INP, SSTa, CHAa
3	0.339	INV, INP, SSTa
4	0.338	INV, INP, SSTa, RAIa
5	0.334	INV, INP, SSTa, CHAa, RAIa

Abbreviations as in Table 2.

## Discussion

It has been suggested that patterns of marine species over large spatial scales are not explained by one single factor but by the combination of several causes and mechanisms [65], [66]. In general, assemblage patterns over large spatial scales may be explained by (1) the biogeographic context in which taxonomic composition is determined by dispersal and disturbance-colonization dynamics [67], (2) models that predict uniform diversity patterns [68], and (3) environmental models that relate biodiversity fluctuations to environmental drivers, including human induced changes [27]. NaGISA-based results on rocky intertidal assemblages showed differences in taxonomic structure among different LMEs, indicating that these assemblages are not homogenous over large spatial scales and refuting the idea of uniformity in assemblage patterns at such scales [28], therefore, we will focus our discussion in the biogeographic context and the environmental drivers.

### The Biogeographic Context

Marine biodiversity is not equally distributed across the globe and species-rich areas may not coincide for different taxonomic groups [69] and/or habitat type (coastal vs. oceanographic) [70]. Our results revealed that gastropod species diversity is especially high in the Mediterranean and the Gulf of Alaska in comparison to other LMEs sampled. The high taxon richness in gastropods suggests that these two LMEs could be considered “hotspots” for gastropod diversity (*sensu* Clarke & Crame [25]). In this sense, both the Mediterranean [71] and the North-Pacific Ocean [72] have previously been reported as regions of high mollusk diversity. In the Mediterranean, its unique geological and biogeographic history, combined with its particular physical and ecological features, have been interpreted as factors resulting in this marine biodiversity hotspot [73]. The relatively substantial biodiversity seen in the Gulf of Alaska could be related to a long evolutionary history of species colonization and further speciation due to isolation within the diverse habitats and a complex spatial heterogeneity that characterizes this Gulf [74].

A relationship between species richness and latitude has been observed in many terrestrial and some marine groups [22], [41], [65], [75]–[78]. In mollusks, these latitudinal trends are variable depending on the hemisphere and the ocean. For example, the diversity of shallow-water species in the northern hemisphere declines toward higher latitudes in both the Pacific and Atlantic coasts, while on the Pacific coast of South America diversity remains constant and relatively low at intermediate latitudes and increases toward higher latitudes (south of 42°S) [44]. It has been hypothesized that this biodiversity peak in the Pacific above 42°S is related to several factors: an increase in shelf area at this latitude, the geographic isolation due to the divergence of major oceanic currents, and the existence of refugia during glaciations which favored speciation and radiation. In contrast, radiation may have been limited on the narrow continental shelves at 10°–42° S [44]. For bivalves, latitudinal and longitudinal gradients exist but are not symmetric between the northern and the southern hemispheres, and a biodiversity hotspot is observed in the Australian provinces in the southern hemisphere [45]. Similarly, an asymmetry has been observed in the diversity of prosobranch gastropods in the eastern Pacific coast from Alaska (70°N) to Cape Horn (55°S) [41], [44]. In the eastern Pacific coast, the highest diversity of prosobranch species occurs between 0°–30° N, decreasing towards higher latitudes in the northern hemisphere, remaining relatively low between 20°–40° S, but increasing towards the pole from 42°S [41], [44]. While our data in intertidal gastropods showed biodiversity hotspots, no clear pattern of species richness in relation to latitude, was observed. In part this may be due to important gaps in our data in terms of small sampling size (which may underestimate taxon richness), geographic cover (lack of data from known species-rich regions such as Australia and in general poor coverage of the southern hemisphere) and the fact that our work is restricted to the shallow-water rocky shore ecosystem. It is possible that especially for these shallow-water (intertidal and down to 10 m depth) assemblages, local variability and patterns override any latitudinal trends that have previously been observed for deeper-water mollusk assemblages. Our results may, therefore, provide support for the hypotheses that different shallow-water taxa may be structured differently along latitude [28] and/or that high species richness may be contained within regional diversity hotspots [29], [70], [79]. For example, analyses for other taxa based on data from the NaGISA project also showed no clear latitudinal pattern of species richness in intertidal rocky shore assemblages of macroalgae and macrofauna (mostly colonial organisms) [28], but have suggested some particular latitudinal trends for macroalgae in the northern hemisphere [30], small intertidal echinoderms [29], and decapods [31].

As with gastropod taxon richness, no latitudinal pattern in gastropod abundance was evident in the present study. The two sites that showed highest gastropod densities (within the Scotian Shelf and Agulhas Current LMEs) were dominated by a single species of Littorinidae, *Littorina littorea* and *Afrolittorina africana*, respectively, both known to numerically dominate vast areas of rocky shores [80]–[82]. However, other sites within these same LMEs did not present high densities of these same gastropods species or within the same genera. The Gulf of Alaska was also characterized by the high abundance of the widely distributed littorinid species *Lacuna vincta*. Numerical dominance of a single species at some sites suggests that some local (small scale) features or processes could be regulating these patterns of high abundances. For example, gastropod density in the rocky intertidal has been reported to be directly related to a local feature such as habitat structure and complexity [83].

Herbivory is key in early succession stages [84] as well as in regulating biodiversity in rocky shore assemblages [85], [86]. Among all the LMEs considered in this study, more than 50% of the gastropod species were herbivores. Warmer regions such as the Kuroshio Current, Mediterranean Sea and Caribbean Sea seemed to have more diversity in feeding habits (despite a large number of species with unknown feeding habits in the Caribbean Sea), which was also the case for the colder Northeast US Coastal Shelf. In contrast, some mid to high-latitude LMEs are characterized nearly exclusively by herbivorous gastropods (Scotian Shelf, Celtic-Biscay Shelf and Agulhas Current) or where they represent more than 90% (Beaufort Sea, Patagonian Shelf, Gulf of Alaska). Such dominance in herbivores and in high densities could be related to high macroalgal biomass particularly between 45° to 60° N [30]. For example, all of the highly abundant species described above are herbivores, and the sites where they occur are also known to have high macroalgal biomass (e.g., 3.28 kg/m^2^ macroalgae for Canso in the Scotian Shelf, 21.38 kg/m^2^ macroalgae for Old Harbor in the Gulf of Alaska, Konar et al., 2010; 14.2 kg/m^2^ macroalgae for Oyster Bay in the Agulhas Current site, Angela Mead, NaGISA unpublished data).

Despite the high family diversity found across LMEs, relatively few of them have wide distributions (less than 10% of the families found in four or more LMEs), indicating that assemblages across regions are quite distinct even at the family level. For example, the Rissoidea, which had the highest species diversity in the overall dataset, were only found in the Gulf of Alaska, the Mediterranean, and the Caribbean Sea. A review of the diversity patterns of this family in the Atlantic and Mediterranean region suggested that the main source regions of speciation for this family are the Mediterranean and the Caribbean, along with the Canaries/Madeira and Cape Verde archipelagos [87]. Hence, high diversity in our study coincided with the suggested speciation centers. Generally, the most common gastropods that graze in rocky shores are true limpets (e.g., Patellidae and Lottiidae within the Patellogastropoda), key-hole limpets (Fissurellidae within the Vetigastropoda), periwinkles (Littorinidae within the Caenogastropoda), and topshells (Trochidae within the Vetigastropoda) [88]. In our study, only Littorinidae, Trochidae, and Fissurellidae were found to be widely distributed, while the relatively high species diversity of Patellidae was restricted to the Mediterranean, the Benguela Current, and the Celtic-Biscay Shelf, and the Lottidae were restricted to the Gulf of Alaska, the Kuroshio Current, and the Scotian Shelf. The distribution patterns of these common families in our study correlate well with their known general biogeographic patterns and phylogenetic history [81], [89]–[93].

In addition to phylogenetic history, these family patterns may also be related to the reproductive mode and dispersal capacity as these may influence gene flow and speciation [94]. In bivalves, a latitudinal diversity gradient from the tropics towards higher latitudes is correlated with larval developmental modes, with planktotrophy (supposedly wider disperal range) dominating the tropics while non-planktotrophy (limited dispersal) increases towards the poles [95]. A review of the reproductive modes characterizing the eight widely distributed families found in this study indicates that all have a wide diversity of reproductive strategies with representatives having planktonic larvae or undergoing direct development (Table S2). Hence, we did not see a close relationship between reproductive mode and wide distribution. Improving our knowledge of the reproductive modes of all rocky shore gastropod species will certainly be helpful to better understand their distribution and global biodiversity but cannot be used alone to explain patterns in biogeography. For example, within the genus *Littorina*, despite extensive knowledge on the reproductive mode of species and a robust species level phylogeny, it is still not clear how non-planktotrophic development evolved [94]. This limits our understanding of how dispersal and gene flow restrictions are responsible for the biogeography of the group.

### The Environmental Model

Environmental variables, either natural or anthropogenic, have been reported to explain species richness or composition in some taxonomic groups using NaGISA data [27]–[29], [31]. Given these previous results with the same database, and the fact that gastropod species richness has been correlated either directly or inversely with sea surface temperature [41], [44] it was surprising to find no significant correlation between environmental and biotic parameters with our data. This could be due however to the fact that we combined the subtidal and intertidal assemblages. In this sense, temperature (SST) would affect more the intertidal than the subtidal assemblages, whereas inorganics (INP) and chlorophyl-a (CHAa) may affect more the subtidal than the intertidal assemblages. SST has been suggested to play an important role in the distribution patterns of rocky shore assemblages [28], [96] as climatic warming may cause changes in species abundance and geographic range [97]. As global warming continues to accelerate [98] and nutrient input to increase, it is expected that anomalies in SST and CHA concentration will become more common; and may consequently alter the structure and functioning of rocky shore gastropod assemblages. The effect of INP may affect the gastropod assemblage either directly by altering organism survival, growth and reproduction [99]–[101], or indirectly by altering the primary producer food sources with consequences in shifts in community structure, diversity, and abundance particularly of the largely abundant herbivores [99], [102], [103]. Pollution was ranked as one of the two most important threats to biodiversity across 25 globally distributed oceanic regions, followed by invasive species and altered temperature [46]. In this work, the perceived correlation with invasive species (INV) likely derives from the fact that some high diversity regions in this study also are known to have high invasive species incidence. The Mediterranean, for example, found in this study to be a rocky shore gastropod hotspot, is also known to be a region with the highest number of marine introduced species (n = 637), of which more than 30% are mollusks [73]. This is three times the total number of introduced species, and four times as many mollusk introduced species, as found in the European Atlantic, a region ranked as second in invasive species [46]. In the Agulhas Current site, the invasive bivalve *Mytilus galloprovincialis* has proliferated along the coast since 1979, and is now the dominant mussel to be found within the low/lower and upper-mid intertidal zones [104]–[106]. It forms a complex three-dimensional matrix in comparison to the native mussels, which provides biogenic habitat for juvenile gastropods but marginalizes adult gastropods, specifically limpets [107]. In parallel, sea temperatures have cooled at a rate of 1°C per decade, whereas air temperatures have increased [108]–[112]. Another impact of the spread of the *M. galloprovincialis* mussel beds has been to minimize bare rock surface where algal holdfasts can attach (personal observation, Angela Mead). This may have impacted the biomass of algae. Interestingly, it is the crustose algal forms that now dominate in these areas [109].

The NaGISA database has several limitations, some of which have already been discussed [31]. In this particular case, the first is the restricted geographic coverage: it sampled less than 20% of the 64 LMEs leaving un-sampled areas of high gastropod diversity (e.g. Australia). The second is the heterogeneity of the sampling efforts. This imposed the need to carry out diversity standardizations to a relatively low number of samples (n = 10 in this case), therefore “loosing” information from well sampled sites. A third limitation, is the fact that for highly diverse areas, the number of samples was simply not enough to collect rare species. Despite these constraints, we still were able to provide a large scale view of rocky shore gastropod diversity and abundance, identify hotspots, dominant species, and widely dispersed families, and explore the environmental parameters which may drive these assemblages. This experience encourages the continuation of large scale research and monitoring activities and initiate them in currently un-sampled localities. One of the largest NaGISA follow-up initiatives at the regional level is the creation of the South American Research Group in Coastal Ecosystems (SARCE). This initiative will continue to assess marine diversity and biomass, and the monitoring of rocky shore ecosystems, with an improved protocol in more than 50 localities in South America while studying ecosystem function and human impacts. Another initiative that will use NaGISA based sampling protocols is the Monitoring Sites 1000 Project conducted by the Ministry of Environment in Japan implemented in 2008 to assess climate change through ecosystem monitoring at 1000 sites including coastal rocky shores throughout Japan for 100 years (http://japan.wetlands.org). Finally, in order to improve our understanding of the role that species have within their assemblages and, therefore, the services that they provide to the ecosystem, these and other similar programs would also benefit from incorporating field experiments on species interactions such as predation, competition and herbivory, as well as observations on recruitment at the global level.

## Supporting Information

Table S1 **List of gastropod species and taxa found at each LME.**
(DOC)

Table S2 **Number of reported species according to the World Register of Marine Species (WoRMS:** http://www.marinespecies.org/ **) and developmental mode of the eight widely distributed families (found in 4 or more LMEs) reported in this paper.** Reported hatching modes: planktotrophic larva (PL), pediveliger (PV), lecithotrophic larva (LL), crawling juvenile (CJ), vivipary (V).
(DOC)
