# Supplementary material for: Large-Scale Spatial Distribution Patterns of Gastropod Assemblages in Rocky Shores
Source: PLoS One. 2013 Aug 13;8(8):e71396. doi: 10.1371/journal.pone.0071396 (PMC3742765; doi:10.1371/journal.pone.0071396)
Supplement: Table S1 — List of gastropod species and taxa found at each LME. (DOC) [file pone.0071396.s001.doc]

**Table S1.** List of gastropod species and taxa found at each LME.

Gulf of Alaska

| *Acmaea mitra* | *Crepipatella sp* | *Lottia cf. digitalis* | *Olivella baetica* |
| --- | --- | --- | --- |
| *Acteocina harpa* | *Cryptobranchia* | *Lottia cf. pelta* | *Onchidella borealis* |
| *Adalaria proxima* | *Cryptobranchia alba* | *Lottia digitalis* | *Onoba carpenteri* |
| *Alia gausapata* | *Cryptobranchia concentrica* | *Lottia instabilis* | *Onoba cerinella* |
| *Alvania sanjuanensis* | *Diaphana* | *Lottia ochracea* | *Puncturella* |
| *Amphissa columbiana* | Eulimidae | *Lottia pelta* | *Puncturella cooperi* |
| *Archidoris sp* | *Fusitriton oregonensis* | *Lottia triangularis* | *Puncturella cucullata* |
| *Astyris gausapata* | Gastropoda | Lottiidae | *Puncturella galeata* |
| *Balcis* | *Granulina margaritula* | *Margarites beringensis* | *Puncturella multistriata* |
| *Boreotrophon clathratus* | *Hermaea vancouverensis* | *Margarites cf. marginatus* | Rissoidae |
| *Boreotrophon multicostatus* | *Homalopoma subobsoletum* | *Margarites marginatus* | Sacoglossa |
| *Boreotrophon stuarti* | *Lacuna* | *Margarites pupillus* | *Scabrotrophon maltzani* |
| *Buccinum baerii* | *Lacuna marmorata* | *Margarites sp* | *Siphonaria thersites* |
| *Cadlina luteomarginata* | *Lacuna variegata* | Muricidae | *Stylidium eschrichtii* |
| *Caecum crebricinctum* | *Lacuna vincta* | *Nassarius mendicus* | *Tectura* |
| *Calliostoma ligatum* | *Limacina* | *Nucella* | *Tectura cf. fenestra* |
| Cerithiidae | *Lirabuccinum dirum* | *Nucella canaliculata* | *Tectura cf. scutum* |
| *Cerithiopsis stejnegeri* | *Lirularia lirulata* | *Nucella lamellosa* | *Tectura persona* |
| *cf. Odostomia* | *Lirularia succincta* | *Nucella lima* | *Tectura scutum* |
| *Cingula katherinae* | *Littorina cf. aleutica* | Nudibranchia | *Trichotropis* |
| *Collisella triangularis* | *Littorina scutulata* | *Ocinebrina interfossa* | *Trichotropis cancellata* |
| *Crepidula nummaria* | *Littorina sitkana* | *Odostomia* | *Trichotropis insignis* |
| *Crepidula sp* | *Lottia* | *Oenopota* | Trochidae |
| *Crepipatella dorsata* | *Lottia borealis* | *Oenopota levidensis* | *Velutina* |
| *Crepipatella lingulata* | *Lottia cf. borealis* | *Oenopota tabulata* | *Vitrinella columbiana* |

Caribbean Sea

| *Aclis sp.*  *Acmaea antillarum*  *Alvania auberiana*  *Anachis sparsa*  *Arene sp*  *Assiminea succinea*  *Barleeia unifasciata*  *Bittiolum varium*  *Bittium varium*  *Caecum antillarum*  *Meioceras cornucopiae (=Caecum cornucopia)*  *Caecum pulchellum*  *Calliostoma sp* | *Retilaskeya emersonii*  *(=Cerithiopsis emersoni)*  *Cerithiopsis greenii*  *Cerithium litteratum*  *Cittarium pica*  *Cittarium sp*  *Cosmioconcha sp*  *Cyclostremiscus cf. jeannae*  *Diodora meta*  *Diodora minuta*  *Epitonium sp.*  *Fissurella barbadensis*  *Fissurella cf. nodosa*  *Fissurella sp.* | *Haminoea elegans*  *Littorina meleagris*  *Littoraria nebulosa (=Littorina nebulosa)*  *Echinolittorina ziczac (=Littorina ziczac)*  *Mitra nodulosa*  *Mitrella ocellata*  *Mitrella sp*  *Nerita peloronta*  *Nerita tessellata*  *Nudibranchia*  *Angiola lineata (=Planaxis lineatus)*  *Supplanaxis nucleus (=Planaxis nucleus)* | *Plicopurpura patula (=Purpura patula)*  *Sinezona confusa*  *Siphonaria sp*  *Stramonita (haemastoma) floridana*  *Stramonita rustica*  *Eulithidium affine (=Tricolia affinis)*  *Tricolia sp.*  Vermetidae  *Zebina browniana*  *Zebina cf. browniana* |
| --- | --- | --- | --- |

Mediterranean Sea

| *Aegires leuckarti* | *Cerithiopsis tubercularis* | *Fissurella nubecula* | *Monophorus thiriotae* |
| --- | --- | --- | --- |
| *Aglaja tricolorata* | *Cerithium rupestre* | *Fossarus ambiguus* | *Nassarius corniculus* |
| *Alvania cimex* | *Chrysallida emaciata* | *Gibberula hilippi* | *Nassarius incrassatus* |
| *Alvania discors* | *Chrysallida intermixta* | *Gibberula miliaria* | *Nodulus contortus* |
| *Alvania lineata* | *Chrysallida obtusa* | *Gibbula ardens* | *Ocinebrina aciculata* |
| *Alvania pagodula* | *Clanculus cruciatus* | *Gibbula racketti* | *Ocinebrina edwardsii* |
| *Alvania subcrenulata* | *Columbella rustica* | *Gibbula turbinoides* | *Odostomella doliolum* |
| *Alvania tenera* | *Conus mediterraneus* | *Haminoea hydatis* | *Odostomia conoidea* |
| *Ammonicera fischeriana* | *Coralliophila meyendorffii* | *Jujubinus exasperatus* | *Odostomia eulimoides* |
| *Aplysia parvula* | *Crisilla semistriata* | *Leufroyia concinna* | *Odostomia turrita* |
| *Ascobulla fragilis* | *Crisilla simulans* | *Mangelia multilineolata* | *Omalogyra atomus* |
| *Barleeia unifasciata* | *Cuthona genovae* | *Mangelia unifasciata* | *Osilinus turbinatus* |
| *Bittium latreilli* | *Cylichnina laevisculpta* | *Mangelia vauquelini* | *Patella caerulea* |
| *Bittium reticulatum* | *Dendropoma petraeum* | *Manzonia crassa* | *Patella rustica* |
| *Bulla striata* | *Diodora gibberula* | *Marshallora adversa* | *Patella ulyssiponensis* |
| *Caecum antillarum* | *Diodora graeca* | *Melaraphe neritoides* | *Philine catena* |
| *Caecum subannulatum* | *Eatonina fulgida* | *Metaxia metaxa* | *Pisinna glabrata* |
| *Caecum trachea* | *Elysia timida* | *Mitrella scripta* | *Pusillina philippi* |
| *Cerithiopsis minima* | *Elysia viridis* | *Monophorus erythrosoma* | *Raphitoma laviae* |
| *Cerithiopsis scalaris* | *Epitonium commune* | *Monophorus perversus* | *Raphitoma linearis* |

| *Raphitoma purpurea* | *Rissoina bruguieri* | *Skeneopsis planorbis* | *Vermetus triquetrus* |
| --- | --- | --- | --- |
| *Retusa truncatula* | *Runcina ferruginea* | *Stramonita haemastoma* | *Vexillum savignyi* |
| *Rissoa guerinii* | *Scissurella costata* | *Tricolia pullus pullus* | *Vexillum tricolor* |
| *Rissoa similis* | *Serpulorbis arenaria* | *Tritonia manicata* | *Vitreolina philippi* |
| *Rissoa ventricosa* | *Setia turriculata* | *Vermetus granulatus* | *Weinkauffia turgidula* |

**Agulhas Current**

| *Afrolittorina africana*  *Afrolittorina knysnaensis* | *Gibbula multicolor* | *Helcion pruinosus* | *Oxystele variegata* |
| --- | --- | --- | --- |

**Benguela Current**

| *Afrolittorina africana* | *Fissurella mutabilis* | *Homalopoma quantillum quantillum* | *Scutellastra argenvillei (=Patella argenvillei)* |
| --- | --- | --- | --- |
| *Burnupena cincta* | *Gibbula cicer* | *Nucella cingulata* | *Scutellastra cochlear (=Patella cochlear)* |
| *Burnupena lagenaria* | *Gibbula multicolor* | *Nucella dubia* | *Scutellastra granularis (=Patella granularis)* |
| *Crepidula porcellana* | *Haliotis midae* | *Nucella squamosa* | *Scutellastra longicosta (=Patella longicosta)* |
| *Cymbula granatina* | *Helcion dunkeri* | *Onchidella capensis* | *Siphonaria capensis* |
| *Cymbula miniata* | *Helcion pectunculus* | *Oxystele tigrina* |  |
| *Cymbula oculus* | *Helcion pruinosus* | *Oxystele variegata* |  |

Scotian Shelf

| *Astyris lunata*  *Buccinum undatum*  *Calliostoma occidentale*  *Crepidula fornicata* | *Euspira heros*  *Lacuna vincta*  *Littorina littorea*  *Littorina obtusata* | *Littorina saxatilis*  *Littorina sp*  *Nassarius trivittatus*  *Nucella lapillus* | *Onoba aculeus*  *Polinices sp.*  *Skeneopsis planorbis*  *Testudinalia testudinalis (=Tectura testudinalis)* |
| --- | --- | --- | --- |

Kuroshio Current

| Aplysiidae | *Littorina brevicula* | Nassariidae | Philinidae |
| --- | --- | --- | --- |
| *Ascobullidae sp* | *Lottia kogamogai* | *Nipponacmea fuscoviridis* | Pleurobranchaeidae |
| *Cellana grata* | *Lottia lindbergi* | *Nipponacmea schrenckii* | *Siphonaria subatra* |
| *Cerithidae gen.sp.1* | *Lottia tenuisculpta* | *Nodilittorina radiata* | Trochidae |
| *Cerithidae gen.sp.2* | Lottiidae | *Nodilittorina sp* | Turridae |
| *Diala albugo* | Mitridae | *Oscilla* | *Zafra sp* |
| *Diniatys dentifer* | *Monodonta labio* | Patellogastropoda |  |
| *Emarginula* | *Monodonta labio confusa* | *Patelloida pygmaea* |  |

Patagonian Shelf

| *Anachis sertulariarum* | *Fissurella radiosa tixierae* | *Tegula patagonica* |  |
| --- | --- | --- | --- |
| *Epitonium frabrizioi* | *Siphonaria lessoni* | *Trophon geversianus* |  |

**Celtic-Biscay Shelf**

| *Buccinum undatum* | *Littorina littorea* | *Melaraphe neritoides* | *Patella depressa* |
| --- | --- | --- | --- |
| *Gibbula cineraria* | *Littorina obtusata* | *Osilinus lineatus* | *Patella vulgata* |
| *Gibbula umbilicalis* |  |  |  |

**Beaufort Sea**

| *Beringius* | *Dendronotus* | *Margarites olivaceus* | *Oenopota* |
| --- | --- | --- | --- |
| *Beringius behringi* | *Flabellina verrucosa* | *Margarites sp* | *Oenopota artica* |
| *Boreocingula martyni* | Gastropoda | *Neptunea* | *Retusa obtusa* |
| *Bulbus smithii* | *Margarites costalis* | *Neptunea lyrata* | *Volutopsius norwegicus* |
| *Cryptonatica affinis* | *Margarites groenlandicus* | Nudibranchia |  |

**Antarctic**

| Unknown Species A  Gastropoda | Unknown species B  Nudibranchia | *Tritonia challengeriana* |  |
| --- | --- | --- | --- |

**North East US Continental Shelf**

| *Odostomia striata (=Aclis striata)*  *Acmaea rubella*  *(=Erginus rubelllus)*  *Acanthodoris pillosa*  Aeolidioidea  *Onoba mighelsi (=Alvania arenaria)*  *Tachyrhnchus reticulatus (=Alvania areolata)*  *Alvania pseudoareolata*  *Alvania sp*  Archaeogastropoda  *Astyris lunata*  *Buccinum sp.*  *Buccinum undatum*  *Colus spA*  *Colus spB*  *Colus stimpsoni* | *Crepidula fornicata*  *Crucibulum striatum*  Cuthonidae  *Cylichna alba*  *Euspira heros*  *Hydrobia sp.*  *Hydrobia truncata (=Hydrobia minuta)*  *Lacuna pallidula*  *Lacuna pallidula neritoidea*  *Lacuna vincta*  *Lepeta caeca*  *Littorina littorea*  *Littorina obtusata*  *Littorina saxatilis* | *Littorina sp*  *Margarites groenlandicus*  *Margarites helicinus*  *Margarites sp*  *Marsenia glabra*  *Astyris lunata (=Mitrella dissimilis)*  *Molleria costulata*  *Nassarius trivittatus*  *Neptunea lyrata decemcostata*  Nudibranchia  *Odostomia sp*  *Omalogyra atomus*  *Onoba sp*  *Onoba aculeus* | *Onoba mighelsii*  *Puncturella noachina*  *Retusa obtusa*  *Skeneopsis planorbis*  *Solariella obscura*  *Tachyrhynchus erosus*  *Testudinalia testudinalis (=Tectura testudinalis)*  *Turritellopsis stimpsoni*  *Velutina sp*  *Velutina velutina (=Velutina laevigata)*  *Velutina undata* |
| --- | --- | --- | --- |
