# Supplementary material for: Large-Scale Spatial Distribution Patterns of Gastropod Assemblages in Rocky Shores
Source: PLoS One. 2013 Aug 13;8(8):e71396. doi: 10.1371/journal.pone.0071396 (PMC3742765; doi:10.1371/journal.pone.0071396)
Supplement: Table S2 — Number of reported species according to the World Register of Marine Species (WoRMS: http://www.marinespecies.org/ ) and developmental mode of the eight widely distributed families (found in 4 or more LMEs) reported in this paper. Reported hatching modes: planktotrophic larva (PL), pediveliger (PV), lecithotrophic larva (LL), crawling juvenile (CJ), vivipary (V). (DOC) [file pone.0071396.s002.doc]

Table S2. Number of reported species according to the World Register of Marine Species (WoRMS: <http://www.marinespecies.org/>) and developmental mode of the eight widely distributed families (found in 4 or more LMEs) reported in this paper. Reported hatching modes: planktotrophic larva (PL), pediveliger (PV), lecithotrophic larva (LL), crawling juvenile (CJ), vivipary (V)

| **Family** | **Number of species (WoRMS)** | **Reproduction / Development** | **Source** |
| --- | --- | --- | --- |
| Buccinidae | 2118 | Egg capsules attached to substrate. Some species have massive spawning behavior. Hatching: PL, LL, CJ | [1] [2] [3] |
| Calyptraeidae | 214 | Protandric. Egg capsules attached to substrate and brooded by female. Genus *Crepidula* exhibits “social” habits. Hatching: PL, PV, LL, CJ | [4] [5] [6] |
| Cerithiidae | 790 | Egg masses. Hatching: PL, CJ (hatching mode related to steno or euryhaline conditions) | [7] [8] |
| Columbellidae | 1793 | Egg capsules attached to live adult shells. Hatching: PL, CJ (hatching mode related to biogeographic area, the trend is PL in the tropical American Pacific and CJ in the Caribbean) | [9] [10] [11] |
| Fissurellidae | 513 | Gametes released directly to seawater. Development: PL | [12] |
| Littorinidae | 768 | Hatching: PL, CJ, V (hatching mode related to species location in the intertidal, CJ in the littoral, viviparity in the supralittoral, PL observed at all levels). Subfamily Littorininae: most species have pelagic egg capsules | [13] [14] |
| Rissoidae | 1239 | Egg capsules. Hatching: PL, CJ | [15] [16] |
| Trochidae | 855 | Broadcast spawning, egg ribbons, gelatinous masses. Hatching: PL, CJ | [17] |

[1] Miloslavich, P. A. & L. Dufresne (1994). Development and effect of female size on egg and juvenile production in the Neogastropod *Buccinum cyaneum* from the Saguenay fjord. Canadian Journal of Fisheries and Aquatic Sciences, 51: 2866-2872.

[2] Miloslavich, P. A. & P. E. Penchaszadeh (1994). Spawn and development of *Engoniophos unicinctus* Say 1825 (Gastropoda Prosobranchia) from the southern Caribbean. The Veliger, 37(4):425-429.

[3] Power, A.J. & B.F. Keegan, 2001. Seasonal patterns in the reproductive activity of the redwhelk, *Neptunea antiqua* (Mollusca: Prosobranchia) in the Irish Sea. Journal of the Marine Biological Association of the United Kingdom 81: 243-250.

[4] Collin, R. 2003. Worldwide patterns in mode of development in calyptraeid gastropods. Marine Ecology Progress Series 247(2): 103-122.

[5] Miloslavich, P., Penchaszadeh, P. E. & A. K. Carbonini. (2003). Embryonic development of *Crepidula aculeata* (Gmelin, 1791) (Caenogastropoda, Calyptraeidae) from the Venezuelan Caribbean. The Veliger, 46(3): 280 – 285.

[6] Miloslavich, P. P. E. Penchaszadeh & E. Klein (2003). Reproduction of *Crepidula navicula* Morch, 1877 and *Crepidula aplysioides* Reeve, 1859 (Caenogastropoda) from Morrocoy and La Restinga lagoon, Venezuela. The Nautilus, 117(4): 121-134.

[7] Houbrick JR. 1970. Reproduction and development in Florida Cerithium. Page 74 in: Annual report of the American Malacological Union, Inc. for 1970.

[8] Cannon LRG. 1975. On the reproductive biology of *Cerithium moniliferum* Kiener (Gastropoda, Cerithiidae) at Heron Island, Great Barrier Reef. Pacific Science 29:353-359.

[9] Fortunato, H., Penchaszadeh, P. & P. Miloslavich. (1998). Spawn and development of *Bifurcium bicanaliferum* from the Central Pacific. The Veliger, 41(2): 208-211.

[10] Fortunato, H. M. 2002. Reproduction and larval development of the *Strombina*-group (Buccinoidea: Columbellidae) and related gastropods: testing the use of the larval shell for inference of development in fossil species. Bolettino Malacologico; 4:111-126

[11] Miloslavich, P., J. M. Díaz, A. K. Carbonini & N. Ardila. 2005. Spawn of *Amphissa* sp. and *Cosmioconcha* sp. (Caenogastropoda: Columbellidae) from the Colombian Caribbean. The Nautilus, 119(4): 157-163.

[12] Collado, G. A. & Donald I. Brown. 2007. Microscopic Anatomy of the Reproductive System in Two Sympatric Species of *Fissurella* Bruguiére, 1789 (Mollusca: Vetigastropoda). Int J. MorphoL, 25(2):315-322.

[13] Mileikovsky, S. A. 1975. Types of larval development in Littorinidae (Gastropoda: Prosobranchia) of the World Ocean, and ecological patterns of their distribution. Marine Biology, 30(2): 129-135.

[14] Reid, D. G. (1989). The comparative morphology, phylogeny and evolution of the gastropod family Littorinidae. Philosophical

Transactions of the Royal Society of London, Series B, 324, 1–110.

[15] Russo, G. F. & F. P. Patti. 2005. Early life history of two closely related gastropods, *Rissoa auriscalpium* and *Rissoa italiensis* (Caenogastropoda: Rissoidae). Marine Biology, 147(2): 429-437, DOI: 10.1007/s00227-005-1586-9.

[16] Avila, S. P., J. Goud, A.M. de Frias Martins. 2012. Patterns of Diversity of the Rissoidae (Mollusca: Gastropoda) in the Atlantic and the Mediterranean Region. The ScientiﬁcWorld Journal, 2012: 1-30, Article ID 164890, doi:10.1100/2012/164890.

[17] Bell, L. J. 1992. Reproduction and Larval Development of the West Indian Topshell, *Cittarium pica* (Trochidae), in the Bahamas. Bulletin of Marine Science, 51(2): 250-266.
